# Traumatic brain injury and risk of early‐onset dementia: A population‐based cohort study

**DOI:** 10.1002/alz.71387

**Published:** 2026-04-14

**Authors:** Aishwarya Saravanan, Katherine Giorgio, Natalia Lakomski, Andrea L. C. Schneider, Barbara A. Olson‐Bullis, Behnam Sabayan, James S. Pankow, Paul S. de Vries, Kristine Yaffe, Alexa Beiser, Abbas Dehghan, Elizabeth A. Peterson, Rachel Zmora, John J. Stephen, Denise Scholtens, Maxwell Mansolf, Lenore J. Launer, Richard F. MacLehose, Pamela L. Lutsey, Norrina B. Allen, Sanaz Sedaghat

**Affiliations:** ^1^ Division of Epidemiology and Community Health University of Minnesota School of Public Health Minneapolis Minnesota USA; ^2^ Department of Biostatistics, Epidemiology and Informatics University of Pennsylvania Perelman School of Medicine Philadelphia Pennsylvania USA; ^3^ HealthPartners Institute Bloomington Minnesota USA; ^4^ Hennepin Healthcare Research Institute Minneapolis Minnesota USA; ^5^ University of Minnesota Medical School Minneapolis Minnesota USA; ^6^ Human Genetics Center, Department of Epidemiology School of Public Health, University of Texas Health Science Center at Houston Houston Texas USA; ^7^ Departments of Psychiatry, Neurology, and Epidemiology University of California San Francisco School of Medicine San Francisco California USA; ^8^ Department of Biostatistics Boston University School of Public Health Boston Massachusetts USA; ^9^ Imperial College London School of Public Health London UK; ^10^ UK Dementia Research Institute London UK; ^11^ Department of Preventive Medicine Northwestern University Feinberg School of Medicine Chicago Illinois USA; ^12^ Department of Medical Social Sciences Northwestern University Feinberg School of Medicine Chicago Illinois USA; ^13^ National Institutes of Health National Institute on Aging Intramural Research Program Baltimore Maryland USA

**Keywords:** early‐onset dementia, late‐onset dementia, mild traumatic brain injury, risk factor, traumatic brain injury

## Abstract

**INTRODUCTION:**

We investigated the association between traumatic brain injury (TBI) and incidence of early‐onset dementia (EOD) versus late‐onset dementia (LOD).

**METHODS:**

We included 501,710 UK Biobank participants (average age 56.5). Over a median 13.6 years of follow‐up, 836 developed EOD (before 65) and 8947 developed LOD (after 65). We estimated hazard ratios (HRs) for TBI's association with EOD and LOD using Cox proportional hazard models with TBI as a time‐varying exposure and coefficient.

**RESULTS:**

TBI was associated with higher risk of both EOD (HR: 4.06 [95% confidence interval: 3.13, 5.26]) and LOD (HR: 2.51 [2.31, 2.72]. Among participants included in both EOD and LOD analyses, the effect estimate was higher for EOD (3.41 vs. 2.80), but the difference was not statistically significant. The association of TBI with EOD was stronger in those with more severe TBIs.

**DISCUSSION:**

TBI is associated with an increased risk of EOD, with a higher risk observed in more severe injuries.

**Highlights:**

- Traumatic brain injury (TBI) is a significant risk factor for early‐onset dementia (EOD).
- TBI was analyzed as a time‐varying exposure and time‐varying coefficient.
- Moderate/severe/penetrating TBIs have a heightened risk of developing EOD.
- TBI is more strongly associated with an earlier age cut‐off for dementia.

## 1 INTRODUCTION

Dementia is a rapidly growing health concern and is a major cause of disability and dependency among older adults worldwide.^1^, ^2^ While most dementia cases are diagnosed in individuals over the age of 65, early‐onset dementia (EOD) is the diagnosis of dementia in individuals younger than 65 years.^3^, ^4^ It presents unique challenges and consequences for patients and takes a toll on the health‐care system, and may present with different etiologies.^4^, ^5^, ^6^

Traumatic brain injury (TBI) has been recognized as a risk factor for dementia.^7^, ^8^, ^9^, ^10^ The mechanisms believed to drive this association include neurovascular injuries and neuro‐inflammation, after which there may be an accumulation of abnormal proteins such as tau and amyloid beta (Aβ). These are typical abnormal proteins seen in several types of dementia.^11^, ^12^, ^13^

While a substantial amount of research has focused on the association between TBI and late‐onset dementia (LOD), there is a relative lack of data about the link between TBI and EOD. Findings from studies examining this link have been inconsistent, possibly due to variations in study design, populations, and definitions of TBI and dementia.^14^, ^15^, ^16^, ^17^, ^18^, ^19^, ^20^, ^21^, ^22^, ^23^, ^24^ In addition, no study has assessed whether the magnitude of the association of TBI with EOD is comparable or even greater than with LOD.

In this study, we aim to investigate the association between TBI and the risk of developing EOD within a population‐based cohort and compare the effect estimates to LOD. We also examine whether these associations differ by TBI severity and sex.

## 2 METHODS

### 2.1 Study population

We used data from the UK Biobank, a large‐scale biomedical database that contains health information from approximately half a million participants aged 40 to 69 years at baseline. Participants were recruited between 2006 and 2010 from the United Kingdom. For this study, we included 501,710 participants from the UK Biobank. This study was approved by the institutional review board (IRB) of the University of Minnesota, IRB Approval Number: STUDY00019630.

### 2.2 TBI

TBI was identified using International Classification of Diseases (ICD)‐9 and ICD‐10 codes (Tables S1 and S2 in supporting information), recorded in the UK Biobank database from hospital inpatient records. These included diagnoses ranging from mild injuries such as concussions and superficial head trauma to moderate and penetrating injuries involving intracranial hemorrhage, skull fractures, traumatic cerebral edema, and open head wounds. The ICD codes for the severity classifications were derived from the Department of Defense's medical classification system, which identified TBI using ICD‐9‐CM and ICD‐10‐CM codes.^25^ Any TBI recorded prior to recruitment into the study and any TBI occurring during the study's follow‐up period before dementia diagnosis was included as an exposure after applying a 1‐year lag period. The earliest recorded case of TBI identified from administrative records occurred in 1980. Self‐reported TBI was ascertained from the conditions coded from the nurse interview portion of the exams. Participants were asked about their past and current medical conditions and the date of diagnosis. Codes for each condition were assigned by the nurse interviewer, or if necessary, after further review by a medical doctor. The code for “head injury” was used for classifying self‐reported TBI.

### 2.3 Dementia incidence

Dementia was ascertained using a combination of self‐reports, hospital records, and death records. The positive predictive value using this method is 82.5%.^26^ Dementia in hospital and death records were identified using ICD‐9 and 10 codes (Tables S3 and S4 in supporting information). For self‐report data, participants were asked if they had a history of “dementia or Alzheimer's or cognitive impairment.” EOD was defined as dementia diagnosed at or before the age of 65 years, and LOD was defined as dementia diagnosed after the age of 65 among those who survived and were dementia free up to the age of 65. The end of the study period differed based on which country the participants were from. For England, the end of the study period was October 31, 2022, for Scotland, it was August 31, 2022, and for Wales, it was May 31, 2022.

### 2.4 Covariates

Covariates included age, sex, race, body mass index (BMI), education, use of antidepressants, hypertension, diabetes, alcohol consumption, and smoking. Of the above covariates, age, sex, race, education, alcohol consumption, and smoking were self‐reported by participants. Race was classified into White participants, Asian participants, Black participants, and other/unknown. BMI was calculated using the height and weight of the participants. Education status was classified into no school/grade school, high school, or technical/vocational/college/graduate/professional. The use of antidepressants was classified into yes or no based on the participant's report of medications currently being taken on a regular basis (Table S5 in supporting information). Data on medication usage were obtained through nurse interviews.

Hypertension was categorized using a combination of systolic and diastolic blood pressure levels and the use of anti‐hypertensive medication. Normal blood pressure was defined as a systolic blood pressure of < 120 mmHg and a diastolic blood pressure of < 80 mmHg without medication use. Elevated blood pressure was indicated by a systolic blood pressure of 120 to 129 mmHg and a diastolic blood pressure of < 80 mmHg, without medication. Hypertension stage 1 was identified with a systolic blood pressure of 130 to 139 mmHg or a diastolic blood pressure of 80 to 89 mmHg or the use of anti‐hypertensive medication. Hypertension stage 2 was defined by systolic blood pressure of ≥ 140 mmHg or diastolic blood pressure of ≥ 90 mmHg, regardless of medication usage.

**RESEARCH IN CONTEXT**

1. **Systematic review**: We conducted a literature review using PubMed, EMBASE, and PsycINFO to identify studies examining the association between traumatic brain injury (TBI) and early‐onset dementia (EOD). While most studies found a positive association between TBI and EOD, a few reported negative or no association. The studies varied in how they defined and ascertained TBI and EOD, their study design, and population characteristics.
2. **Interpretation**: Our study contributes to the literature by investigating the association of TBI with EOD and late‐onset dementia in the same cohort, with a time‐varying exposure. We also explored how the association changes when stratified based on the severity of TBI.
3. **Future directions**: Future research should explore the pathological relationship between TBI and EOD. This could clarify if there is a higher risk of dementia with TBI at younger ages.

Diabetes mellitus was categorized based on non‐fasting plasma glucose, hemoglobin A1c (HbA1c), and medication use. Individuals were classified as not having diabetes if they had a HbA1c < 5.7% or non‐fasting plasma glucose < 140 mg/dL and no anti‐diabetic medication use. Prediabetes was determined by a HbA1c between 5.7% and 6.4% or non‐fasting plasma glucose of 140 to 199 mg/dL and no anti‐diabetic medication use. Diabetes mellitus was determined by HbA1c of ≥ 6.5%, non‐fasting plasma glucose of ≥ 200 mg/dL, or the use of anti‐diabetic medication. Smoking status was categorized into non‐smoker, former smoker, and current smoker. Alcohol consumption was measured in grams per week.

### 2.5 Statistical analysis

We excluded individuals with dementia at baseline (*n* = 230). This resulted in a final study population of 501,710 individuals (Figure S1 in supporting information). A lag period of 1 year between TBI and dementia was incorporated, and participants who had < 1 year of follow‐up were considered unexposed in the analysis. The date of TBI diagnosis was recorded, and for participants with multiple TBIs, the first recorded TBI date was used.

Demographic and clinical characteristics, including sex, race, diabetes mellitus, hypertension, smoking status, education, and use of antidepressants, were summarized using the number and percentage of participants in each category. Age and BMI are described using mean and standard deviation, whereas alcohol consumption is described using the median and interquartile range. This was done using the gtsummary package in R.^27^

Data on covariates were missing for 5.2% (26,009) of participants. We used multiple imputation with chained equations to impute missing values using the mice package in R.^28^ This imputation was applied to all fully adjusted models except for the Fine–Gray model in sensitivity analysis due to computational limitations. In that case, the 5.2% of participants with missing values were excluded from the final model.

For the analysis of EOD, the follow‐up time was censored at the age of 65 in addition to death or the end of the study period. For LOD, follow‐up began after the age of 65 until the earliest dementia diagnosis, death, or end of the study period. Crude incidence rates of EOD and LOD with and without TBI were calculated. In Cox proportional hazard models, TBI was modeled as a time‐varying exposure, and its coefficient was also allowed to vary according to whether the participant was younger or older than 65 in follow‐up. Age was used as the timescale in this model. The time‐varying coefficient in the model allowed us to estimate hazard ratios (HRs) for the association of TBI with EOD and LOD, and to test whether those associations are statistically significantly different. We achieved these results using the survival package in R, following the “Using Time‐Dependent Covariates” vignette.^29^ Time‐varying exposure allowed each participant's exposure status to change over time. A participant was considered unexposed before their first recorded TBI diagnosis and exposed thereafter. This method ensures that only person‐time after the TBI contributes to the exposed risk period, which improves the accuracy of HR estimates.

The HR was estimated using three models. The unadjusted model did not adjust for any confounders. The minimally adjusted model was adjusted for sex and race. The fully adjusted model was adjusted for sex, race, BMI, education, use of antidepressants, diabetes, hypertension, alcohol consumption, and smoking.

To explore whether the severity of TBI changes the effect estimates, we categorized participants into those with mild TBI, moderate TBI, severe TBI, and penetrating TBI using ICD codes (Table S1). Due to the small number of individuals within the moderate, severe, and penetrating TBI categories, they were combined into one category. For participants with more than one TBI incident, the classification was based on the most severe injury. We then conducted stratified analyses according to this TBI severity categorization.

#### 2.5.1 Sensitivity analysis

Given that antidepressant use may be a mediator in the association of TBI and dementia, we re‐fit the fully adjusted model excluding the antidepressant use covariate. We also performed a sensitivity analysis using self‐reported TBI as the exposure. As the age cut‐off for EOD is arbitrary, an analysis was performed in which the cut‐off for dementia diagnosis was varied to 55, 60, 65, 70, 75, 80, and 85 years of age. This was done to compare the HRs of the association of TBI with dementia across varying age cut‐offs. We also performed a sensitivity analysis in which TBI was classified as “no TBI,” “TBI before age 43,” and “TBI at or after age 43” rather than including TBI as a time‐varying exposure. Death is a competing risk for dementia; therefore, we fit Fine–Gray subdistribution hazard models for LOD.

We performed a stratified analysis based on participants’ self‐reported sex. We tested for effect modification by sex by including a multiplicative interaction term between TBI and sex in the fully adjusted Cox proportional hazards models. We performed an additional stratified analysis based on participant age at enrollment. For this we grouped participants as < 55, 55 to 65, or 65+ at the start of follow‐up due to differences in the time at risk contributed to the EOD and LOD time periods for these groups. Participants in the 55 to 65 age category are the most representative group because they are both young enough to have incident EOD after the start of follow‐up and old enough to have incident LOD before the end of follow‐up.

## 3 RESULTS

### 3.1 Demographic characteristics

The study sample included 501,710 participants from the UK Biobank, with 16,959 individuals having a history of TBI and 484,751 without TBI. Table S6 in supporting information shows the number of people with TBIs by dementia status and by age of enrollment into the study. The demographic and clinical characteristics of the study population are presented in Table 1. The TBI population had a higher proportion of males (56%) compared to the non‐TBI population (45%). The mean age was higher in the TBI group (57.9 years) compared to the non‐TBI group (56.5 years). Racial distribution was similar across both groups, with the majority being White (95% in TBI vs. 94% in non‐TBI). The TBI group had a higher percentage of diabetes (8% vs. 5%) and higher rates of smoking and antidepressant use compared to the non‐TBI group.

**TABLE 1:** Characteristic features of the whole study population and a comparison based on TBI status.

Characteristic	TBI population (*n *= 16,959)	No TBI population (*n* = 484,751)	Overall population (*N* = 501,710)
Sex (male), *n* (%)	9549 (56%)	219,223 (45%)	228,722 (46%)
Age (years), mean (SD)	57.9 (8.22)	56.5 (8.09)	56.5 (8.09)
Race, *n* (%)			
White	16,181 (95%)	455,779 (94%)	471,960 (94%)
Asian	292 (1.7%)	11,141 (2.3%)	11,433 (2.3%)
Black	156 (0.9%)	7891 (1.6%)	8047 (1.6%)
Other/unknown	330 (1.9%)	9940 (2.1%)	10,270 (2.0%)
BMI, mean (SD)	27.7 (5.03)	27.4 (4.79)	27.4 (4.80)
Diabetes, *n* (%)			
Normal	12,515 (76%)	381,262 (81%)	393,777 (81%)
Pre‐diabetes	2535 (15%)	66,214 (14%)	68,749 (14%)
Diabetes	1328 (8%)	23,635 (5%)	24,963 (5%)
Hypertension, *n* (%)			
Normal blood pressure	1868 (11%)	66,340 (14%)	68,208(14%)
Elevated blood pressure	1519 (9%)	51,199 (11%)	52,718 (11%)
Hypertension stage 1	5082 (30%)	142,320 (30%)	147,402 (30%)
Hypertension stage 2	8252 (49%)	219,498 (46%)	227,750 (46%)
Smoking status, *n* (%)			
Non‐smoker	8148 (48%)	264,963 (55%)	273,111 (55%)
Former smoker	5923 (35%)	166,822 (35%)	172,745 (35%)
Current smoker	2752 (16%)	50,157 (10%)	52,909 (11%)
Education status, *n* (%)			
No school/ grade school	4120 (24%)	81,797 (17%)	85,917 (17%)
High school	3891 (23%)	112,927 (23%)	116,818 (23%)
Technical/ vocational/ college/ graduate/ professional	8902 (53%)	289,203 (60%)	298,105 (60%)
Alcohol consumption (grams/week), median (IQR)	56 (0, 144)	48 (2, 112)	48 (2, 112)
Use of antidepressants, *n* (%)			
Yes	1832 (11%)	30,912 (6.4%)	32,744 (6.5%)
No	15,127 (89%)	453,839 (94%)	468,966 (93%)

*Note*: Number of participants with missing values for the following variables: BMI: 3099, diabetes: 14,221, hypertension: 5632, smoking status: 2945, education status: 870, alcohol consumption: 9027.

Abbreviations: BMI, body mass index; IQR, interquartile range; SD, standard deviation; TBI, traumatic brain injury.

### 3.2 Incidence rates of dementia

The median [interquartile range] follow‐up time was 10.00 [5.00–13.30] years for EOD and 8.47 [4.65–11.95] years for LOD. The crude incidence rates of EOD and LOD are shown in Table 2. The incidence rate of EOD was higher in the TBI population (9.35 per 10,000 person‐years; 95% confidence interval [CI]: 7.17–11.98) compared to the non‐TBI population (2.02 per 10,000 person‐years; 95% CI: 1.88–2.17). Similarly, the incidence rate of LOD was also higher in the TBI population (112.52 per 10,000 person‐years; 95% CI: 104.07–121.48) compared to the non‐TBI population (30.88 per 10,000 person‐years; 95% CI: 30.22–31.52).

**TABLE 2:** Incidence rates of EOD and LOD with and without TBI.

	Incidence rate – with TBI (per 10,000 person years)		Incidence rate – without TBI (per 10,000 person years)	
	Measure	95% CI	Measure	95% CI
EOD	9.35	7.17–11.98	2.02	1.88–2.17
LOD	112.52	104.07–121.48	30.88	30.22–31.55

Abbreviations: CI, confidence interval; EOD, early‐onset dementia; LOD, late‐onset dementia; TBI, traumatic brain injury.

### 3.3 TBI and EOD and LOD

Table 3 presents the results of the Cox regression analysis of TBI and its association with EOD and LOD. In the unadjusted model, TBI was associated with a significantly higher risk of both EOD (HR: 4.74; 95% CI: 3.66–6.14) and LOD (HR: 2.74; 95% CI: 2.53–2.96). These associations remained significant after adjusting for potential confounders in both the minimally adjusted and the fully adjusted model. The fully adjusted model showed a HR of 4.06 (95% CI: 3.13–5.26) for EOD and 2.51 (95% CI: 2.31–2.72) for LOD. The difference in HRs between EOD and LOD was statistically significant (*p* value < 0.05) in all models.

**TABLE 3:** Comparison of HRs for the association of TBI with EOD and LOD.

Models	EOD (ncase/*N* = 836/427,947)	LOD (ncase/*N* = 8947/336,540)	EOD vs. LOD
	HR (95% CI)	HR (95% CI)	*p* value
Unadjusted model	4.74 (3.66–6.14)	2.74 (2.53–2.96)	<0.05
Minimally adjusted model^a^	4.58 (3.54–5.94)	2.71 (2.50–2.94)	<0.05
Fully adjusted model^b^	4.06 (3.13–5.26)	2.51 (2.31–2.72)	<0.05

Abbreviations: CI, confidence interval; EOD, early‐onset dementia; HR, hazard ratio; LOD, late‐onset dementia; TBI, traumatic brain injury.

a Adjusted for sex and race.

^b^ Adjusted for sex, race, body mass index, education status, use of antidepressants, hypertension, diabetes, alcohol, and smoking.

Table 4 displays the association of TBI with EOD and LOD, stratified by different severities of TBI. In the fully adjusted model, individuals with mild TBI had a HR of 3.94 (95% CI: 2.92–5.33) for EOD and 2.54 (95% CI: 2.32–2.78) for LOD. Those with moderate/severe/penetrating TBI had an even higher HR of 6.22 (95% CI: 3.66–10.57) for EOD and 3.52 (95% CI: 2.97–4.18) for LOD.

**TABLE 4:** Comparison of HRs for association of TBI with EOD and LOD by severity of TBI.

TBI categorized based on severity	EOD	LOD	EOD vs. LOD
	HR (95% CI)	HR (95% CI)	*p* value
Unadjusted model			
Mild	4.60 (3.41 – 6.21)	2.78 (2.54–3.05)	<0.05
Moderate/ severe/ penetrating	7.31 (4.31–12.41)	3.83 (3.23–4.54)	0.02
Minimally adjusted model^a^			
Mild	4.46 (3.30–6.03)	2.77 (2.53–3.03)	<0.05
Moderate/ severe/ penetrating	7.01 (4.13–11.91)	3.74 (3.15–4.43)	0.03
Fully adjusted model^b^			
Mild	3.94 (2.92–5.33)	2.54 (2.32–2.78)	<0.05
Moderate/ severe/ penetrating	6.22 (3.66–10.57)	3.52 (2.97–4.18)	<0.05

*Note*: *N* cases / *N*: mild TBI estimates: EOD: 798/425,085; LOD: 8549/333,667; Moderate/severe/penetrating TBI estimates: EOD: 685/414,866; LOD: 7215/323,446

Abbreviations: CI, confidence interval; EOD, early‐onset dementia; HR, hazard ratio; LOD, late‐onset dementia; TBI, traumatic brain injury.

a Adjusted for sex and race.

^b^ Adjusted for sex, race, body mass index, education status, use of antidepressants, hypertension, diabetes, alcohol, and smoking.

### 3.4 Sensitivity analysis

Removing antidepressant use from the covariates did not change the findings (EOD HR: 4.28, 95% CI 3.30–5.54; LOD HR: 2.60, 95% CI 2.40–2.81). Self‐reported TBI was associated with a higher hazard of both EOD and LOD (EOD HR: 2.50, 95% CI: 1.30–4.82; LOD HR: 1.73, 95% CI: 1.22–2.47; Table S7 in supporting information). We did not observe a significant interaction between TBI and sex in relation to EOD risk in the fully adjusted model (*p* value: 0.20) or LOD risk (*p* value: 0.997; Table S8 in supporting information). The subdistribution HR for the association between TBI and LOD was 2.39 (95% CI: 2.19–2.60) after full adjustment (Table S9 in supporting information).

**FIGURE 1:**
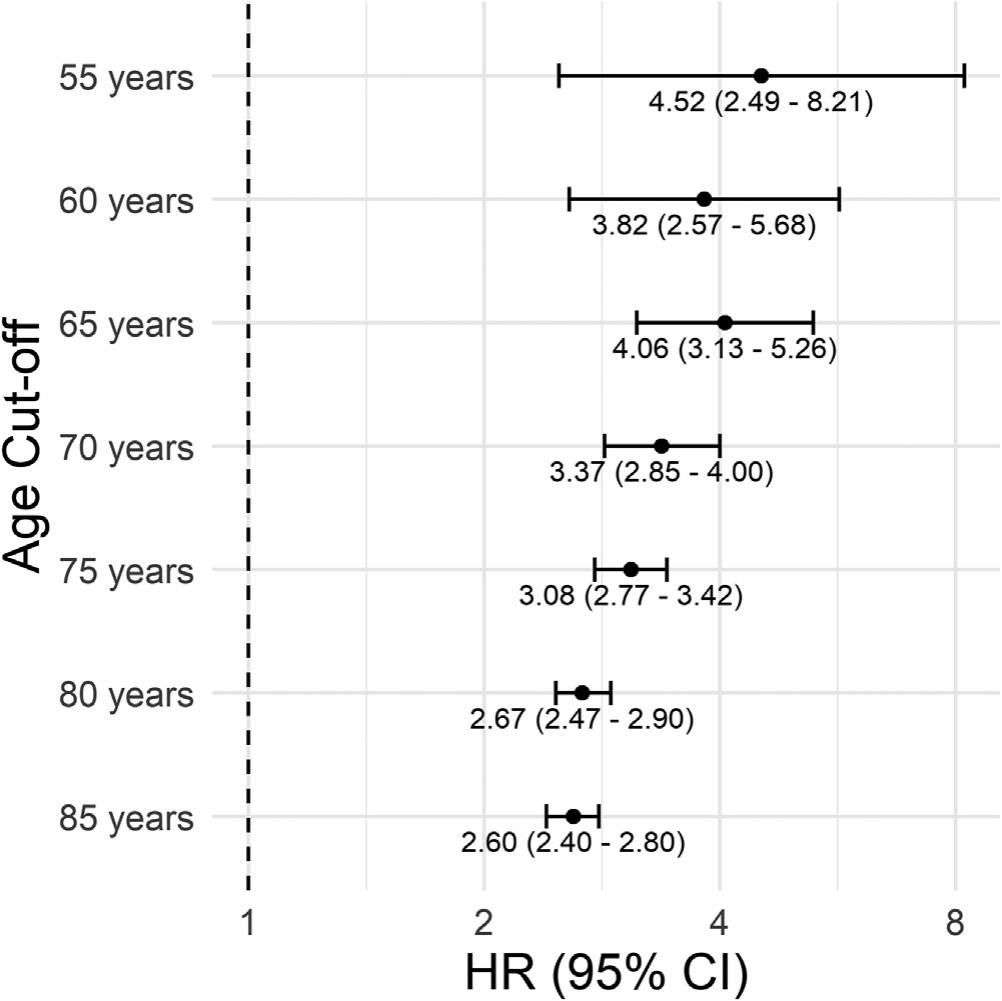
Forest plot of HRs for the association of TBI with dementia, by varying age cut‐offs. CI, confidence interval; HR, hazard ratio; TBI, traumatic brain injury.

Figure 1 and Table S10 in supporting information present the HRs for dementia with varying age cut‐offs (55, 60, 65, 70, 75, 80, and 85 years). The fully adjusted HRs ranged from 4.52 (95% CI: 2.49–8.21) for age cut‐off 55 to 2.60 (95% CI: 2.40–2.80) for age cut‐off 85. The association between TBI and dementia remained significant across different age thresholds, although the strength of the association decreased as the age cut‐off increased. Categorizing based on age at TBI instead of including TBI as a time‐varying exposure, we observed that TBIs occurring earlier in life appear more strongly related to EOD than TBIs occurring later in life (Table S11 in supporting information). However, we did not observe the same trend for LOD. When stratified by age at enrollment, we observed larger effect estimates for EOD than LOD across all age groups (Table S12 in supporting information). However, in the middle group (ages 55–65, including individuals who contributed comparably to both EOD and LOD analyses), the effect estimates for EOD and LOD were more similar compared to the main findings and the difference between the effect estimates for EOD and LOD was not statistically significant (*p* value: 0.41; Table S12).

## 4 DISCUSSION

In this population‐based cohort, we found that experiencing a TBI is associated with a higher risk of both EOD and LOD (among those who survived and are dementia free through the age of 65). When including individuals who contributed comparably to both EOD and LOD analyses, the effect estimate was stronger for EOD; however, the difference between EOD and LOD effect estimates did not reach statistical significance. The stratified analysis showed that more severe TBIs are associated with a greater risk of EOD.

TBI triggers a series of neurobiological events such as axonal damage, blood–brain barrier disruptions, and neuroinflammation, which may last beyond the initial trauma.^11^, ^12^ These events are key in the accumulation of proteins such as Aβ and tau, which are usually seen in dementia cases.^11^, ^30^ This chronic neuroinflammation, along with oxidative stress and synaptic dysfunction, contributes to the progressive cognitive decline observed in dementia.^31^, ^32^, ^33^, ^34^ To our knowledge, only one study has explored the pathological relationship between TBI and EOD. This case series analyzed brain tissue from veterans who had been diagnosed with EOD after TBI and found *post mortem* neuropathological findings such as tau proteins, amyloid plaques, and white matter injuries.^35^

The relationship between TBI and dementia, particularly EOD, has been supported in multiple studies.^14^, ^15^, ^16^, ^17^, ^18^, ^19^, ^22^, ^23^, ^24^, ^36^, ^37^, ^38^, ^39^ While some studies reported a positive association between TBI and EOD,^14^, ^15^, ^16^, ^17^, ^18^, ^19^, ^22^, ^23^ others reported negative or no association.^24^, ^36^ The inconsistency may have arisen from variations in study design, population characteristics, TBI severity, or the criteria used to define TBI and dementia.

Studies that did not find an association between TBI and EOD relied on self‐reported head injuries.^24^, ^36^ Self‐reported head injuries have the potential for recall bias and could weaken the strength of the association.^40^ This observation aligns with our findings, for which the strength of association was weaker when self‐reported injury data was used. A recent study using the UK Biobank aimed to investigate risk factors for EOD. While TBI was among the risk factors examined, no effect estimates were reported due to the absence of TBI cases among individuals with EOD.^41^ This may be because the study only considered participants who had experienced TBI at baseline. In the present study, we included all individuals who experienced TBI throughout the follow‐up and used a time‐varying approach.

We observed a stronger association between TBI and EOD compared to LOD and also when using different age cutoffs, with the strongest association at a cutoff of 55 (HR: 4.52) and the weakest at 85 (HR: 2.60). Our findings are in line with a nationwide Swedish database study, which reported that the association of TBI with dementia risk decreased over time, though it remained notable even after 30 years.^42^ Several reasons can explain this finding. First, the brain may be more vulnerable to developing dementia earlier after the injury rather than later in life.^43^, ^44^, ^45^ Second, cases with earlier onset may be more directly tied to the neurological damage caused by the trauma, often with greater severity.^17^ Third, younger individuals may have fewer age‐related cognitive declines. This could make the contribution of TBI to dementia more pronounced at a younger age compared to later in life, when other factors, such as age‐related neurodegeneration, may also play a significant role.^38^ Fourth, the differences in effect estimates may partially reflect variations in age at enrollment because the ability to experience EOD versus LOD is structurally tied to baseline age and length of follow‐up. The youngest participants did not reach age 65 during follow‐up and were therefore excluded from the LOD group, while the oldest participants were already older than 65 at baseline and were not included in the EOD group. In sensitivity analyses restricted to participants with the potential to contribute to both time periods (55–65 at enrollment), the difference in the association between TBI and EOD versus LOD was attenuated, which suggests the strength of the association in younger onset dementia can be partially explained by the differences in age of enrollment. We also examined the relationship between early versus late TBI with dementia and found that the association between TBIs occurring earlier in life (before 43) appear more strongly related to EOD than TBIs occurring later in life (after 43). However, we did not observe the same trend for LOD.

A major strength of this study is the direct comparison of effect estimates for EOD and LOD within the same population, which reveals variations in TBI's impact based on the dementia onset period. Additionally, by modeling TBI as a time‐varying exposure, we accounted for when the TBI occurred. This approach helps avoid immortal time bias, which can underestimate risk by misclassifying unexposed time as exposed, leading to more accurate estimates of TBI's effect on dementia.^46^, ^47^

However, there are also limitations to consider. The observational nature of the cohort study means that causality cannot be established. Despite our efforts to adjust for confounders, residual confounding may still exist. Another limitation is the use of ICD codes for defining dementia and TBI, which could influence the accuracy of diagnosis and result in misclassification.^26^, ^48^, ^49^ Additionally, dementia ascertainment from death records may introduce misclassification. However, only 3% of EOD and 4% of LOD cases were based exclusively on death records. Given that the earliest data for diagnosis of TBI is from 1980, TBIs occurring early in life would not be captured for the older UK Biobank participants. This likely diluted the observed association with LOD. Additionally, because outcome categorization is contingent on age, LOD cases only include those who survived and stayed dementia free through age 65. Furthermore, death is a competing risk for dementia, particularly at older ages. However, the association of TBI and LOD did not change when the competing risk of death was included in Fine–Gray models. Due to the small number of TBIs, we grouped moderate, severe, and penetrating TBIs into a single category, which prevents us from assessing differences in dementia risk between these severities. While the UK Biobank offers a large cohort, it is important to acknowledge that it is not fully representative of the general population. The cohort is predominantly White (94%), with limited racial and ethnic diversity. This makes it difficult to examine how the relationship between TBI and dementia may vary across racial or ethnic groups. There may also be selection bias with healthier and more socioeconomically advantaged individuals being more likely to enroll than the sampling population.^50^

This study provides evidence that TBI is a significant risk factor for both EOD and LOD (among those who survived and are dementia free through the age of 65). Given the observed association between TBI and EOD, clinicians should be vigilant in monitoring cognitive function in patients with a history of TBI, particularly those who have sustained moderate to severe injuries. Early detection and intervention strategies could be crucial in mitigating the progression of cognitive decline in these individuals.

## CONFLICT OF INTEREST STATEMENT

The authors declare no conflicts of interest. Author disclosures are available in the supporting information.

## CONSENT STATEMENT

All participants provided informed consent for their participation in the UK Biobank study, from which these data were obtained.

## Supporting information




**Table S1**: ICD‐10 codes used to define TBI and TBI severity
**Table S2**: ICD‐9 codes used to define TBI and TBI severity.
**Table S3**: ICD‐10 codes used to define dementia
**Table S4**: ICD‐9 codes used to define dementia
**Table S5**: Medications used for the classification of variable: Use of antidepressants
**Table S6**: Dementia outcome status by TBI and age of enrollment into UKBB
**Table S7**: Comparison of HRs for the association of self‐reported TBI with EOD and LOD
**Table S8**: Comparison of HRs for the association of TBI with EOD and LOD ‐ stratified by sex
**Table S9**: Subdistribution HRs for the association of TBI with LOD, in the context of the competing risk of death
**Table S10**: HRs for the association of TBI with dementia, by varying age cut‐offs
**Table S11**: Comparison of HRs for the association of TBI with EOD and LOD, with TBI categorized as occurring before or after age 43, relative to having no TBI
**Table S12**: Comparison of HRs for the association of TBI with EOD and LOD, by age at baseline
**Figure S1**: Flowchart of cohort selection

Supporting Information
