# Supplementary material for: Traumatic brain injury and risk of early‐onset dementia: A population‐based cohort study
Source: Alzheimers Dement. 2026 Apr 14;22(4):e71387. doi: 10.1002/alz.71387 (PMC13079064; doi:10.1002/alz.71387)
Supplement: Supplementary file 1 — Table S1: ICD‐10 codes used to define TBI and TBI severity Table S2: ICD‐9 codes used to define TBI and TBI severity. Table S3: ICD‐10 codes used to define dementia Table S4: ICD‐9 codes used to define dementia Table S5: Medications used for the classification of variable: Use of antidepressants Table S6: Dementia outcome status by TBI and age of enrollment into UKBB Table S7: Comparison of HRs for the association of self‐reported TBI with EOD and LOD Table S8: Comparison of HRs for the association of TBI with EOD and LOD ‐ stratified by sex Table S9: Subdistribution HRs for the association of TBI with LOD, in the context of the competing risk of death Table S10: HRs for the association of TBI with dementia, by varying age cut‐offs Table S11: Comparison of HRs for the association of TBI with EOD and LOD, with TBI categorized as occurring before or after age 43, relative to having no TBI Table S12: Comparison of HRs for the association of TBI with EOD and LOD, by age at baseline Figure S1: Flowchart of cohort selection [file ALZ-22-e71387-s002.docx]

**Supplementary Tables and Figures:**

**Table S1**: ICD-10 codes used to define TBI and TBI severity.

| **ICD-10 Codes** | **Definition** | **Severity classification** |
| --- | --- | --- |
| F07.2 | Postconcussional syndrome | Mild |
| S00 | Superficial injury of head | Mild |
| S00.0 | Superficial injury of scalp | Mild |
| S00.7 | Multiple superficial injuries of head | Mild |
| S00.8 | Superficial injury of other parts of head | Mild |
| S00.9 | Superficial injury of head, part unspecified | Mild |
| S01 | Open wound of head | Mild |
| S01.0 | Open wound of scalp | Mild |
| S01.7 | Multiple open wounds of head | Mild |
| S01.8 | Open wound of other parts of head | Mild |
| S01.9 | Open wound of head, part unspecified | Mild |
| S02 | Fracture of skull and facial bones | Mild |
| S02.0 | Fracture of vault of skull | Moderate |
| S02.00 | Fracture of vault of skull (closed) | Moderate |
| S02.01 | Fracture of vault of skull (open) | Penetrating |
| S02.1 | Fracture of base of skull | Moderate |
| S02.10 | Fracture of base of skull (closed) | Moderate |
| S02.11 | Fracture of base of skull (open) | Penetrating |
| S02.2 | Fracture of nasal bones | Mild |
| S02.3 | Fracture of orbital floor | Mild |
| S02.4 | Fracture of malar and maxillary bones | Mild |
| S02.5 | Fracture of tooth | Mild |
| S02.6 | Fracture of mandible | Mild |
| S02.7 | Multiple fractures involving skull and facial bones | Mild |
| S02.8 | Fractures of other skull and facial bones | Mild |
| S02.9 | Fracture of skull and facial bones, part unspecified | Mild |
| S04.0 | Injury of optic nerve and pathways | Severe |
| S06 | Intracranial injury | Moderate |
| S06.0 | Concussion | Mild |
| S06.00 | Concussion (without open intracranial wound) | Mild |
| S06.01 | Concussion (with open intracranial wound) | Penetrating |
| S06.1 | Traumatic cerebral oedema | Moderate |
| S06.10 | Traumatic cerebral oedema (without open intracranial wound) | Moderate |
| S06.2 | Diffuse brain injury | Moderate |
| S06.20 | Diffuse brain injury (without open intracranial wound) | Moderate |
| S06.21 | Diffuse brain injury (with open intracranial wound) | Penetrating |
| S06.3 | Focal brain injury | Moderate |
| S06.30 | Focal brain injury (without open intracranial wound) | Moderate |
| S06.31 | Focal brain injury (with open intracranial wound) | Penetrating |
| S06.4 | Epidural hemorrhage | Moderate |
| S06.40 | Epidural hemorrhage (without open intracranial wound) | Moderate |
| S06.41 | Epidural hemorrhage (with open intracranial wound) | Penetrating |
| S06.5 | Traumatic subdural hemorrhage | Moderate |
| S06.50 | Traumatic subdural hemorrhage (without open intracranial ound) | Moderate |
| S06.51 | Traumatic subdural hemorrhage (with open intracranial wound) | Penetrating |
| S06.6 | Traumatic subarachnoid hemorrhage | Moderate |
| S06.60 | Traumatic subarachnoid hemorrhage (without open intracranial wound) | Moderate |
| S06.61 | Traumatic subarachnoid hemorrhage (with open intracranial wound) | Penetrating |
| S06.7 | Intracranial injury with prolonged coma | Severe |
| S06.70 | Intracranial injury with prolonged coma (without open intracranial wound) | Severe |
| S06.8 | Other intracranial injuries | Moderate |
| S06.80 | Other intracranial injuries (without open intracranial wound) | Moderate |
| S06.81 | Other intracranial injuries (with open intracranial wound) | Penetrating |
| S06.9 | Intracranial injury, unspecified | Moderate |
| S06.90 | Intracranial injury, unspecified (without open intracranial wound) | Moderate |
| S06.91 | Intracranial injury, unspecified (with open intracranial wound) | Penetrating |
| S07 | Crushing injury of head | Moderate |
| S07.0 | Crushing injury of face | Mild |
| S07.9 | Crushing injury of head, part unspecified | Mild |
| S08 | Traumatic amputation of part of head | Moderate |
| S08.0 | Avulsion of scalp | Mild |
| S08.1 | Traumatic amputation of ear | Mild |
| S08.8 | Traumatic amputation of other parts of head | Mild |
| S08.9 | Traumatic amputation of unspecified part of head | Mild |
| S09 | Other and unspecified injuries of head | Mild |
| S09.0 | Injury of blood vessels of head, not elsewhere classified | Moderate |
| S09.1 | Injury of muscle and tendon of head | Mild |
| S09.2 | Traumatic rupture of ear drum | Mild |
| S09.7 | Multiple injuries of head | Moderate |
| S09.8 | Other specified injuries of head | Mild |
| S09.9 | Unspecified injury of head | Mild |

**Table S2:** ICD-9 codes used to define TBI and TBI severity.

| **ICD-9 Codes** | **Definition** | **Severity classification** |
| --- | --- | --- |
| 3102 | Post concussional syndrome | Mild |
| 800 | Fracture of vault of skull | Moderate |
| 8000 | Fracture of vault of skull - closed without mention of intracranial injury | Moderate |
| 8001 | Fracture of vault of skull - closed with intracranial injury | Moderate |
| 8002 | Fracture of vault of skull - open without mention of intracranial injury | Penetrating |
| 8003 | Fracture of vault of skull - open with intracranial injury | Penetrating |
| 801 | Fracture of base of skull | Moderate |
| 8010 | Fracture of base of skull - closed without mention of intracranial injury | Moderate |
| 8011 | Fracture of base of skull - closed with intracranial injury | Moderate |
| 802 | Fracture of face bones | Mild |
| 8020 | Fracture of nasal bones, closed | Mild |
| 8022 | Fracture of mandible, closed | Mild |
| 8023 | Fracture of mandible, open | Mild |
| 8024 | Fracture of malar and maxillary bones, closed | Mild |
| 8026 | Fracture of orbital floor (blow-out), closed | Mild |
| 8028 | Fracture of other facial bones, closed | Mild |
| 803 | Other and unqualified skull fractures | Mild |
| 8030 | Other, unqualified skull fractures. - closed without mention of intracranial injury | Mild |
| 8031 | Other and unqualified skull fractures - closed with intracranial injury | Moderate |
| 850 | Concussion | Mild |
| 8509 | Concussion | Mild |
| 851 | Cerebral laceration and contusion | Moderate |
| 8510 | Cerebral laceration or contusion without open intracranial wound | Moderate |
| 852 | Subarachnoid, subdural and extradural hemorrhage, following injury | Moderate |
| 8520 | Subarachnoid or subdural or extradural hemorrhage. following injury without open intracranial wound | Moderate |
| 853 | Other and unspecified intracranial hemorrhage following injury | Moderate |
| 8530 | Other or unspecified intracranial hemorrhage following injury open intracranial wound | Moderate |
| 854 | Intracranial injury of other and unspecified nature | Moderate |
| 8540 | Other or unspecified intracranial injury without open intracranial wound | Moderate |
| 8541 | Other or unspecified intracranial injury with open intracranial wound | Penetrating |
| 873 | Other open wound of head | Mild |
| 8730 | Open wound of scalp, without mention of complication | Mild |
| 8738 | Other and unspecified open wound of head without mention of complication | Mild |
| 8738 | Other and unspecified open wound of head without mention of complication | Mild |
| 9070 | Late effect of intracranial injury without mention of skull fracture | Mild |

**Table S3:** ICD-10 codes used to define dementia

| **ICD 10 Code** | **ICD 10 Text** |
| --- | --- |
| A81.0 | Sporadic Creutzfeldt-Jakob disease |
| F00 | Dementia in Alzheimer's disease |
| F00.0 | Dementia in Alzheimer's disease with early onset |
| F00.1 | Dementia in Alzheimer's disease with late onset |
| F00.2 | Dementia in Alzheimer's disease, atypical or mixed type |
| F00.9 | Dementia in Alzheimer's disease, unspecified |
| F01 | Vascular dementia |
| F01.0 | Vascular dementia of acute onset |
| F01.1 | Multi-infarct dementia |
| F01.2 | Subcortical vascular dementia |
| F01.3 | Mixed cortical and sub-cortical vascular dementia |
| F01.8 | Other vascular dementia |
| F01.9 | Vascular dementia, unspecified |
| F02 | Dementia in other diseases classified elsewhere |
| F02.0 | Dementia in Picks disease |
| F02.1 | Dementia in Creutzfeldt-Jacob disease |
| F02.2 | Dementia in Huntington’s disease |
| F02.3 | Dementia in Parkinson’s disease |
| F02.4 | Dementia in HIV disease |
| F02.8 | Dementia in other specified diseases classified elsewhere |
| F03 | Unspecified dementia |
| F05.1 | Delirium superimposed on dementia |
| F10.6 | Mental and behavioral disorders due to use of alcohol - amnesic syndrome |
| G30 | Alzheimer’s disease |
| G30.0 | Alzheimer’s disease with early onset |
| G30.1 | Alzheimer’s disease with late onset |
| G30.8 | Other Alzheimer's disease |
| G30.9 | Alzheimer's disease unspecified |
| G31.0 | Circumscribed brain atrophy |
| G31.1 | Senile degeneration of brain |
| G31.8 | Other specified degenerative diseases of nervous system |
| I67.3 | Binswanger’s disease |

**Table S4:** ICD-9 codes used to define dementia

| **ICD-9 Codes** | **Definition** |
| --- | --- |
| 290.2 | Senile dementia, depressed or paranoid type |
| 290.3 | Senile dementia with acute confusional state |
| 290.4 | Arteriosclerotic dementia |
| 291.2 | Other alcoholic dementia |
| 294.1 | Dementia in other conditions classified elsewhere |
| 331.0 | Alzheimer's disease |
| 331.1 | Pick's disease |
| 331.2 | Senile degeneration of brain |
| 331.5 | Creutzfeldt-Jakob disease |

**Table S5:** Medications used for the classification of variable: Use of antidepressants

| **Serial number** | **Medication** |
| --- | --- |
| 1 | Doxepin |
| 2 | Sertraline |
| 3 | Paroxetine |
| 4 | Amitriptyline+Chlordiazepoxide 12.5mg/5mg Capsule |
| 5 | Amitriptyline Hydrochloride+Perphenazine 10mg/2mg Tablet |
| 6 | Fluoxetine |
| 7 | Fluvoxamine |
| 8 | Amitriptyline |
| 9 | Dosulepin |
| 10 | Venlafaxine |
| 11 | Efexor 37.5mg Tablet |
| 12 | Citalopram |
| 13 | Mirtazapine |
| 14 | Bupropion |
| 15 | Escitalopram |
| 16 | Duloxetine |

**Table S6:** Dementia outcome status by TBI and age of enrollment into UKBB

| **Age of enrollment**  (max. age at end of follow-up) |  | **EOD**  **N (%*)** | **LOD**  **N (%*)** | **No dementia**  **N (%*)** |
| --- | --- | --- | --- | --- |
| **< 55**  (70.6) | no TBI | 397 (90%) | 35 (95%) | 187,839 (97%) |
|  | TBI | 42 (10%) | 2 (5%) | 5,530 (3%) |
|  | *TOTAL* | *439 (100%)* | *37 (100%)* | *193,369 (100%)* |
| **55 – 65**  (81.6) | no TBI | 377 (95%) | 3,860 (93%) | 222,182 (97%) |
|  | TBI | 20 (5%) | 304 (7%) | 7,359 (3%) |
|  | *TOTAL* | *397 (100%)* | *4,164 (100%)* | *229,541 (100%)* |
| **66 +**  (87.9) | no TBI | NA | 4,398 (93%) | 65,663 (95%) |
|  | TBI | NA | 348 (7%) | 3,354 (5%) |
|  | *TOTAL* | *NA* | *4,746 (100%)* | *69,017 (100%)* |

TBI - Traumatic Brain Injury, EOD - Early-onset Dementia, LOD - Late-onset Dementia

**Table S7**: Comparison of HRs for the association of self-reported TBI with EOD and LOD

| **Models** | **EOD**  **(ncase/N = 836/427,947)** | **LOD**  **(ncase/N = 8,947/336,540)** | **EOD vs LOD** |
| --- | --- | --- | --- |
|  | *HR*  *(95% CI)* | *HR*  *(95% CI)* | *p-value* |
| *Unadjusted model* | 2.76  (1.43 - 5.33) | 1.68  (1.28 - 2.21) | 0.17 |
| *Minimally adjusted model** | 2.64  (1.37 – 5.10) | 1.63  (1.22 - 2.11) | 0.17 |
| *Fully adjusted model^†^* | 2.50  (1.30 – 4.82) | 1.57  (1.20 - 2.06) | 0.20 |

EOD - Early-onset Dementia, LOD - Late-onset Dementia, HR - Hazard Ratio, CI - Confidence Interval

*Adjusted for sex and race

^†^Adjusted for sex, race, body mass index, education status, use of antidepressants, hypertension, diabetes, alcohol, and smoking.

**Table S8:** Comparison of HRs for the association of TBI with EOD and LOD - stratified by sex

| **Models** | **EOD**  **(ncase/N = 836/427,947)** | **LOD**  **(ncase/N = 8,947/336,540)** | **EOD vs LOD** |
| --- | --- | --- | --- |
|  | *HR*  *(95% CI)* | *HR*  *(95% CI)* | *p - value* |
| **Male** | **(ncase/N = 452/192,293)** | **(ncase/N = 4,643/154,048)** |  |
| *Unadjusted model* | 4.81  (3.54 - 6.53) | 2.75  (2.48 - 3.06) | <0.05 |
| *Minimally adjusted model** | 4.84  (3.57 - 6.57) | 2.76  (2.48 - 3.07) | <0.05 |
| *Fully adjusted model*^†^ | 4.24  (3.12 - 5.76) | 2.53  (2.27 - 2.81) | <0.05 |
| **Female** | **(ncase/N = 384/235,654)** | **(ncase/N = 4,304/182,492)** |  |
| *Unadjusted model* | 3.75  (2.27 - 6.18) | 2.65  (2.35 - 2.99) | 0.19 |
| *Minimally adjusted model** | 3.75  (2.27 - 6.19) | 2.66  (2.36 - 3.00) | 0.19 |
| *Fully adjusted model*^†^ | 3.33  (2.02 - 5.50) | 2.47  (2.18 - 2.78) | 0.25 |

TBI - Traumatic Brain Injury, EOD - Early-onset Dementia, LOD - Late-onset Dementia, HR -Hazard Ratio, CI - Confidence Interval

p-value for interaction (fully adjusted model) for TBI with sex - EOD: 0.20; LOD: 0.997

*****Adjusted for race

^†^Adjusted for race, body mass index, education status, use of antidepressants, hypertension, diabetes, alcohol, and smoking.

**Table S9:** Subdistribution HRs for the association of TBI with LOD, in the context of the competing risk of death

| **Models** | **LOD**  **n dementia = 8,947**  **n death = 28,200**  **N = 336,540** |
| --- | --- |
|  | *SHR (95% CI)* |
| *Unadjusted model* | 2.57  (2.37 - 2.79) |
| *Minimally adjusted model** | 2.55  (2.35 - 2.76) |
| *Fully adjusted model^†^* | 2.39  (2.19 – 2.60) |

TBI - Traumatic Brain Injury, LOD - Late-onset Dementia, SHR – Subdistribution Hazard Ratio, CI - Confidence Interval

*****Adjusted for sex and race

^†^Adjusted for sex, race, body mass index, education status, use of antidepressants, hypertension, diabetes, alcohol, and smoking. 5.2% of the sample was excluded due to missing values of covariates.

**Table S10**: HRs for the association of TBI with dementia, by varying age cut-offs

| **AGE CUT OFF** | **Ncase/N** | **MODELS** | **DEMENTIA** |
| --- | --- | --- | --- |
|  |  |  | *HR (95% CI)* |
| 55 years | 121/210,662 | Unadjusted model | 6.56 (3.69 – 11.66) |
|  |  | Minimally adjusted model* | 6.53 (3.65 – 11.68) |
|  |  | Fully adjusted model^†^ | 4.52 (2.49 – 8.21) |
| 60 years | 316/308,877 | Unadjusted model | 5.36 (3.64 – 7.90) |
|  |  | Minimally adjusted model | 5.12 (3.47 – 7.58) |
|  |  | Fully adjusted model | 3.82 (2.57 – 5.68) |
| 65 years | 836/427,947 | Unadjusted model | 4.74 (3.66 – 6.14) |
|  |  | Minimally adjusted model | 4.58 (3.54 – 5.94) |
|  |  | Fully adjusted model | 4.06 (3.13 – 5.26) |
| 70 years | 2,238/501,703 | Unadjusted model | 4.12 (3.48 – 4.86) |
|  |  | Minimally adjusted model | 3.94 (3.33 – 4.66) |
|  |  | Fully adjusted model | 3.37 (2.85 – 4.00) |
| 75 years | 5,546/501,710 | Unadjusted model | 3.49 (3.14 – 3.88) |
|  |  | Minimally adjusted model | 3.42 (3.08 – 3.80) |
|  |  | Fully adjusted model | 3.08 (2.77 – 3.42) |
| 80 years | 9,088/501,710 | Unadjusted model | 2.96 (2.73 – 3.20) |
|  |  | Minimally adjusted model | 2.92 (2.70 – 3.16) |
|  |  | Fully adjusted model | 2.67 (2.47 – 2.90) |
| 85 years | 9,783/501,710 | Unadjusted model | 2.84 (2.63 – 3.07) |
|  |  | Minimally adjusted model | 2.81 (2.61 – 3.04) |
|  |  | Fully adjusted model | 2.60 (2.40 – 2.80) |

TBI - Traumatic Brain Injury, HR -Hazard Ratio, CI - Confidence Interval

*****Adjusted for sex and race

^†^Adjusted for sex, race, body mass index, education status, use of antidepressants, hypertension, diabetes, alcohol, and smoking.

**Table S11:** Comparison of HRs for the association of TBI with EOD and LOD, with TBI categorized as occurring before or after age 43, relative to having no TBI

| **Models** | **TBI age of exposure** | **EOD**  **(ncase/N = 836/427,947)** | **LOD**  **(ncase/N = 8,947/336,540)** | **EOD vs LOD** |
| --- | --- | --- | --- | --- |
|  |  | *HR (95% CI)* | *HR (95% CI)* | *p - value* |
| *Unadjusted model* | no TBI | (ref) | (ref) | (ref) |
|  | TBI < 43 | 4.09  (2.25 – 7.44) | 1.15  (0.37 – 3.57) | 0.052 |
|  | TBI 43 + | 2.39  (1.80 – 3.17) | 1.71  (1.58 – 1.85) | <0.05 |
| *Minimally adjusted model** | no TBI | (ref) | (ref) | (ref) |
|  | TBI < 43 | 3.87  (2.13 – 7.04) | 1.10  (0.35 – 3.41) | 0.054 |
|  | TBI 43 + | 2.34  (1.77 – 3.11) | 1.70  (1.57 – 1.84) | <0.05 |
| *Fully adjusted model^†^* | no TBI | (ref) | (ref) | (ref) |
|  | TBI < 43 | 3.40  (1.87 – 6.18) | 0.93  (0.30 – 2.90) | <0.05 |
|  | TBI 43 + | 2.06  (1.55 – 2.73) | 1.56  (1.44 – 1.69) | 0.07 |

TBI - Traumatic Brain Injury, EOD - Early-onset Dementia, LOD - Late-onset Dementia, HR -Hazard Ratio, CI - Confidence Interval

*****Adjusted for sex and race

^†^Adjusted for sex, race, body mass index, education status, use of antidepressants, hypertension, diabetes, alcohol, and smoking.

**Table S12:** Comparison of HRs for the association of TBI with EOD and LOD, by age at baseline

| **Models** | **EOD**  **(ncase/N = 836/427,947)** | **LOD**  **(ncase/N = 8,947/336,540)** | **EOD vs LOD** |
| --- | --- | --- | --- |
|  | *HR (95% CI)* | *HR (95% CI)* | *p - value* |
| **< 55** | **(ncase/N = 439/193,845)** | **(ncase/N = 37/34,227)** |  |
| *Unadjusted model* | 4.85  (3.53 – 6.67) | 2.00  (0.48 – 8.32) | 0.24 |
| *Minimally adjusted model** | 4.54  (3.30 – 6.25) | 1.92  (0.46 – 8.00) | 0.25 |
| *Fully adjusted model^†^* | 3.40  (2.46 – 4.71) | 1.33  (0.32 – 5.55) | 0.21 |
| **55 - 65** | **(ncase/N = 397/234,102)** | **(ncase/N = 4,164/228,550)** |  |
| *Unadjusted model* | 3.99  (2.55 – 6.26) | 3.15  (2.80 – 3.54) | 0.32 |
| *Minimally adjusted model** | 3.86  (2.46 – 6.05) | 3.09  (2.75 – 3.48) | 0.35 |
| *Fully adjusted model^†^* | 3.41  (2.17 – 5.35) | 2.80  (2.49 – 3.15) | 0.41 |
| **66 +** | **(ncase/N = 0/0)** | **(ncase/N = 4,746/73,763)** |  |
| *Unadjusted model* | NA | 2.43  (2.17 – 2.71) | NA |
| *Minimally adjusted model** | NA | 2.41  (2.16 – 2.69) | NA |
| *Fully adjusted model^†^* | NA | 2.28  (2.04 – 2.55) | NA |

TBI - Traumatic Brain Injury, EOD - Early-onset Dementia, LOD - Late-onset Dementia, HR -Hazard Ratio, CI - Confidence Interval

*****Adjusted for sex and race

^†^Adjusted for sex, race, body mass index, education status, use of antidepressants, hypertension, diabetes, alcohol, and smoking.

**Figure S1**: Flowchart of cohort selection

All participants from UK Biobank

(N= 501,940)

Participants with prevalent dementia excluded

(N = 230)

Study population

(N= 501,710)

Those who had TBI (N = 20,034)

Those who did not have TBI (N = 481,676)

Those who had less than 1 year of follow-up were considered unexposed (N = 3,075)

Those who had TBI with at least 1 year of follow-up (N = 16,959)

No TBI or less than 1 year follow-up with TBI (N = 484,751)
